# Poly(ADP-ribosyl)ation and DNA repair synthesis in the extracts of naked mole rat, mouse, and human cells

**DOI:** 10.18632/aging.101959

**Published:** 2019-05-13

**Authors:** Anastasiya A. Kosova, Mikhail M. Kutuzov, Alexei N. Evdokimov, Ekaterina S. Ilina, Ekaterina A. Belousova, Svetlana A. Romanenko, Vladimir A. Trifonov, Svetlana N. Khodyreva, Olga I. Lavrik

**Affiliations:** 1 Institute of Chemical Biology and Fundamental Medicine, Siberian Branch of the Russian Academy of Sciences, Novosibirsk 630090, Russia; 2 Novosibirsk State University, Novosibirsk 630090, Russia; 3 Institute of Molecular and Cellular Biology, Siberian Branch of the Russian Academy of Sciences, Novosibirsk 630090, Russia; * Equal contribution

**Keywords:** base excision repair, Heterocephalus glaber, Mus musculus, poly(ADP‐ribose) polymerases, DNA polymerase

## Abstract

DNA repair capacity in cells of naked mole rat (Hgl), a species known for its longevity and resistance to cancer, is still poorly characterized. Here, using the whole-cell extracts (WCEs) of Hgl, mouse and human cells, we studied the interrelation between DNA synthesis on the substrates of base excision repair and the activity of poly(ADP-ribose) polymerases (PARPs) responsible for the transfer of the ADP-ribose moieties onto different targets. The level of PAR synthesis was more than ten-fold higher in human WCE as compared to rodent WCEs, while the efficiency of DNA synthesis was comparable. Under conditions of PAR synthesis, the efficiency of DNA synthesis was only slightly enhanced in all extracts and in mouse WCEs unusual products of the primer elongation were detected. The results obtained with WCEs, recombinant proteins and recently found ability of PARPs to attach the ADP-ribose moieties to DNA allowed us to attribute these products to primer mono(ADP-ribosyl)ation (MARylation) at the 5ʹ-terminal phosphate by PARP3 during the DNA synthesis. PARP1/PARP2 can then transfer the ADP-ribose moieties onto initial ADP-ribose. Our results suggest that MARylation/PARylation of DNA in the extracts depends on the ratios between PARPs and can be controlled by DNA-binding proteins.

## Introduction

DNA repair systems are considered as a key factor in mammalian cells, which counteracts genomic instability and is associated with aging and oncogenesis. The naked mole rat (*Heterocephalus glaber*, Hgl) is a long‐lived and tumor‐resistant rodent. However until recently DNA repair efficiency in Hgl cells has not been directly tested. Using extracts from Hgl and mouse (Mus musculus, Mmu) fibroblasts we compared the activities of some enzymes involved in base excision repair (BER) [1]. Hgl cell extracts were slightly more efficient compared to Mmu in the removal of the uracil residues and cleavage of the abasic (AP) sites but not in the DNA synthesis on the BER substrates. The level of the poly(ADP-ribose) (PAR) synthesis catalyzed by poly(ADP-ribose) polymerases (PARPs) was also higher in Hgl cell extracts. In addition, Hgl cell extracts contain higher amounts of PARP1 as revealed by cross-linking of the extract proteins to chemically reactive DNA intermediates bearing photoreactive nucleotide analogs or AP sites [1]. Taking into account the known roles of PARPs and their activity in the BER process, we were interested in studying the effect of PARylation on DNA repair synthesis on the specific substrates of BER. The whole-cell extracts, which differ in the efficiency of PAR synthesis, seem to be an attractive model system for this purpose since the ratios of proteins in the extracts reflect their state in real cells.

BER is one of the main strategies of cellular defense against single-base lesions in DNA. The generally accepted BER model in mammalian cells involves two pathways [2,3]. In the so-called short-patch (SP) pathway, a specific DNA glycosylase removes a damaged nucleobase with the formation of an AP site. This site is then incised by apurinic/apyrimidinic endonuclease 1 (APE1), which leads to a nick with the 5ʹ-deoxyribose phosphate (dRP) and 3ʹ-hydroxyl group. DNA polymerase β (Polβ) inserts then one nucleotide and removes the dRP group [2,3]. The modified dRP-groups cannot be removed by the 5ʹ-dRP lyase activity of Polβ and the repair of these blocked intermediates can proceed by the long-patch (LP) BER pathway. In this pathway, DNA synthesis is performed by Polβ or Polδ(ε), and the modified 5ʹ-dRP group is displaced as a part of the 5ʹ-flap structure, which is formed during the strand-displacement DNA synthesis [2,3].

The PARP family of enzymes, which is also referred to ADP-ribosyltransferases (ARTD), includes 17 known members sharing the conserved ADP-ribosyltransferase domain [4]. Most of ARTDs are able to transfer the ADP-ribose monomers onto acceptor proteins or synthesize the ADP-ribose polymer covalently attached to target proteins using nicotinamide adenine dinucleotide (NAD^+^) as a substrate [4]. PARP1, PARP2, and PARP3 can detect DNA damages and couple interaction with DNA to the activation of their own catalytic ability that plays a key role in coordination between the cell response and DNA damage. PARP1 and PARP2 are able to synthesize a PAR polymer, while PARP3 transfers only a single ADP-ribose unit onto targets. These reactions are called poly(ADP-ribosyl)ation (PARylation) and mono(ADP-ribosyl)ation (MARylation), respectively [4]. These PARPs are associated with most of DNA repair pathways including BER [5–13]. We have previously shown that PARP1 and PARP2 differently interact with DNA intermediates of various DNA-dependent processes [6]. Moreover, PARP2 and PARP3 are selectively activated by 5ʹ-phosphorylated DNA strand breaks that suggests PARPs’ involvement in the particular DNA repair pathways [14].

Using a system reconstituted from purified BER proteins, bovine testis nuclear extract and model DNA intermediates of BER, we have previously shown that PARP1 interacts with the central intermediate of BER [15–17] and may act as a regulator of Polβ activity in LP-BER, while its influence on the Polβ gap-filling activity in SP-BER was insignificant. Comparison of the PARP1 and PARP2 effect on DNA repair synthesis by Polβ on the BER substrates revealed that both PARPs inhibit its activity; NAD^+^ can alleviate the inhibitory effect of PARP1 but not of PARP2 [6].

We have recently demonstrated PARylation of the DNA termini by PARP1 and PARP2 *in vitro* [18]. Later, we and others have revealed an ability of PARP3 to mono(ADP-ribosyl)ate DNA termini [19–21]. Moreover, these 5ʹ-end (ADP-ribosyl)ated DNAs are significantly more efficient substrates for PAR chain elongation by purified PARP1 and PARP2 as compared to unmodified DNAs. However, the impact of PARylation of DNA substrates on the efficiency of DNA synthesis has not been addressed so far.

To analyze proteins in the cell extracts involved in the interaction with the BER substrates, we used an affinity labeling approach (aka affinity modification), which is based on the covalent attachment of chemically reactive analogs of substrate/ligand to enzyme/protein. As we have shown earlier, this approach is effective to study the protein-nucleic acid interactions in complex systems, such as cell extracts [22–24]. Introduction of the modifying groups (e.g., mimicking specific DNA lesions), into DNA probes allows one to target them to the proteins of the particular DNA repair pathway [22–24]. The DNA probes used in this work that contain the photoactivatable dCMP derivative represent analogs of the BER substrates.

Here, we evaluated the relative efficiency of PAR synthesis and degradation and DNA synthesis on the BER substrates in the absence or presence of NAD^+^ in Hgl, mouse and human WCEs.

During DNA synthesis in the presence of NAD^+^ the unusual products of primer elongation were found in Mmu cell extract. To evaluate the commonality of the phenomenon, we included in further experiments the extract of 3T3 cells, a widely used mouse cell line. First, the efficiency of PAR synthesis and degradation in the extracts was examined. We also compared in details DNA synthesis on the BER substrates catalyzed by endogenous DNA and the effect of NAD^+^ in the extracts of Hgl, mouse, and human cells. Taking into account the recently found ability of PARPs to attach the ADP-ribose moieties to DNA and the here obtained data concerning the cell extracts and recombinant proteins, we attributed NAD^+^-dependent products to the PARP3-dependent mono(ADP-ribosyl)ation of the primers at the 5ʹ-terminal phosphate during the DNA synthesis. PARP1/PARP2 can then transfer the ADP-ribose moieties onto initial ADP-ribose. We have found for the first time a possibility of coupling of DNA (ADP-ribosyl)ation with DNA synthesis. Our results suggest that MARylation/PARylation of DNA in the extracts depends on the ratios between PARPs and other DNA-binding proteins.

## RESULTS

### Synthesis and degradation of PAR in the extracts

Considering the known role of the system of PAR synthesis/degradation in cell response to DNA damage and DNA repair, we compared the processes of PAR synthesis and degradation in four WCEs. The total level of PAR synthesis was evaluated by two approaches based on the use of [^32^P]NAD^+^ as a substrate. In one way, the aliquots of the reaction mixtures were loaded onto Whatman 1 paper. The paper-bound radioactivity level after the removal of unreacted NAD^+^ reflects the total amount of PAR synthesized by endogenous PARPs. An example of the kinetics of PAR synthesis is shown in Figure S1 A and B. The linear parts of the kinetic curves of this synthesis were observed until 1.5 min for HEK293T WCE and at least for 3 min for rodent WCE. It is important of note a large difference in the rate of PAR synthesis in the extracts of human and rodent cells. This fact prompted us to evaluate the efficiency by an additional approach.

**Figure 1:**
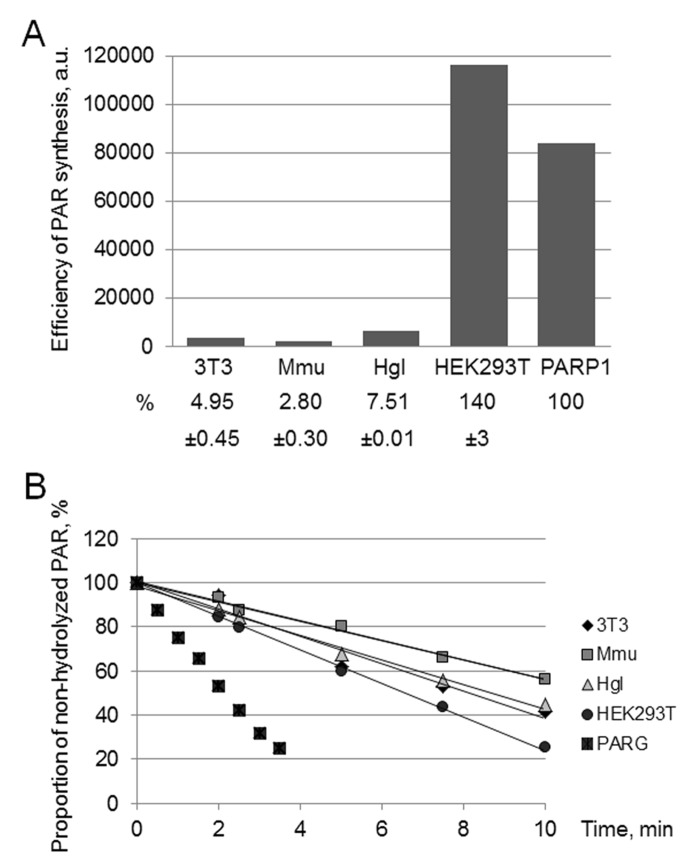
**Efficiency of PAR synthesis (A) and degradation (B) in WCEs.** (**A**) PAR synthesis was performed for 1 min at 37 °C in the reaction mixture containing standard buffer components and 0.6 A_260_/mL activated DNA, 0.5 mg/mL cell extract proteins (or 10 nM recombinant human PARP1), and 20 μM [^32^P]NAD^+^. The reaction mixtures were treated and analyzed as described in the section ‘Synthesis and degradation of PAR in the extracts. PARP activity assay’. The yield of PAR analyzed by SDS-PAGE (the gel is shown in Fig. S1) is represented as a bar chart in arbitrary phosphorimager units. The analysis of PAR synthesis for three independent experiments is shown in numerical form under the bar chart. The data are the mean ± SD. In each experiment, the amount of PAR synthesized in the extract was normalized to that synthesized by 10 nM recombinant PARP1. (**B**) The reaction mixtures containing standard components, [^32^P]PAR synthesized as described in the section ‘Synthesis and degradation of PAR in the extracts. PARP activity assay’, and 0.5 mg/mL cell extract proteins or 10 nM recombinant PARG were incubated at 37 °C for different time intervals. Aliquots were further processed and analyzed as described in the section ‘Synthesis and degradation of PAR in the extracts. PAR degradation assay’. The amount of [^32^P]PAR in an equal aliquot of the control mixture (no proteins added) before incubation was taken as 100%. The points on the experimental curves represent the average of three independent experiments. Standard deviation did not exceed 10%.

Alternatively, the level of PAR synthesis by endogenous PARPs was analyzed by gel electrophoresis. The autoradiograph of the gel demonstrates an example of the analysis of PAR synthesized in WCEs and by PARP1 (Figure S1C). Quantification of the PAR yield in the extracts is shown as a bar chart in Figure 1A. The efficiency of PAR synthesis differs less than 3 times between the rodent cell extracts, with the maximum yield being observed in Hgl WCE. At the same time, the yield of PAR in HEK293T WCE is one order of magnitude higher than that in rodent cell extracts.

The replacement of activated DNA as a cofactor for endogenous PARPs of the extracts by the SP-BER substrate decreased the yield of PAR only by 20% in all extracts (data not shown). The efficient activation of PAR synthesis in the extracts by the BER substrates gives grounds to further study the impact of PARylation on DNA synthesis on these substrates.

To evaluate the rate of PAR degradation in the extracts, we used the following approach. Radioactively labeled PAR was synthesized by human recombinant PARP1. PAR synthesis was stopped by EDTA and the reaction mixture was used as a source of PAR without purification.

Poly(ADP-ribose) glycohydrolase (PARG) is the main enzyme responsible for PAR degradation in mammalian cells [25]. PARG acts as both endo- and exoglycosidase, which releases PAR of different length and ADP-ribose monomers and does not require bivalent metal ions for its activity [25]. The kinetic curves of PAR hydrolysis are shown in Figure 1B. In the negative control (no extract proteins added) the level of PAR decomposition for 30 min was less than 5% (data not shown). Again HEK293T WCE was the most active. In the case of PAR degradation, however, the rates between the extracts differed less than two fold.

The found difference in the activity of the PAR synthesis/degradation system between the WCEs makes them a convenient model system to evaluate a contribution of these processes to the regulation of DNA synthesis in the SP- and LP pathways of BER.

### Effect of (ADP-ribosyl)ation on DNA synthesis

In the previous paper, we have already compared DNA synthesis in the specifically prepared extracts of mouse and naked mole rat fibroblasts on the BER substrates, however, the effect of PARylation on DNA synthesis was not addressed [1]. In contrast to the previous study, we used here the whole-cell extracts without fractionation of proteins (including the extract of human cells) and three BER substrates. They contain a nick and have the common upstream primer bearing the [^32^P]-label at the 5ʹ end, while the downstream oligonucleotides have either the deoxyribose phosphate (dRP) or diethylene glycol phosphate (pDEG) residues at the 5ʹ ends or the single-stranded dangling flap. The 5ʹ dRP moiety was obtained after the removal of the uracil residue by Ung, which led to the formation of the substrate of the initial stage of SP pathway. The DNA duplex containing the pDEG residue at the 5ʹ end of a downstream oligonucleotide mimics the substrate of the initial stage of the LP pathway since the pDEG residue cannot be removed by Polβ in contrast to the 5ʹ-dRP group. The flap-containing DNA can be considered as a product of the strand-displacement DNA synthesis and represents the substrate of the later stages of the LP pathway.

**Figure 2:**
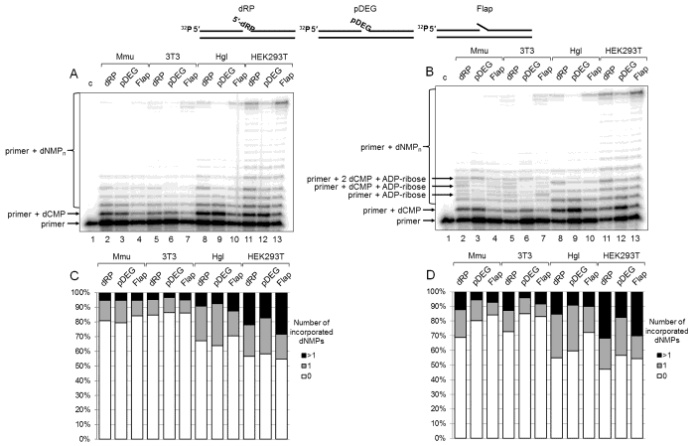
**DNA synthesis and effect of PAR synthesis in WCEs.** (**A**) DNA synthesis in the absence of NAD^+^. The cell extract proteins (0.5 mg/mL) were incubated for 5 min with 100 nM DNA duplexes bearing dRP, pDEG, or flap in the presence of 0.1 mM dNTPs (as described in the section ‘DNA synthesis assay’). (**B**) DNA synthesis in the presence of NAD^+^. The same as in (A), but in the presence of 0.5 mM NAD^+^. The unknown products are marked. Lanes 1 in A and B correspond to the initial primer (control). The types of DNA and cell lines are indicated. (**C** and **D**) Quantification of the products shown in Figure 2A and 2B, respectively. The white parts of the bars correspond to the non‐elongated primer, the grey parts reflect the amount of the primer elongated by one dNMP, and the black parts correspond to the products of strand‐displacement DNA synthesis. The intensity of the products is calculated as a percentage of the total radioactivity in the lane. The structures of DNA substrates are schematically shown at the top.

The results on DNA synthesis in the extracts are shown in Figure 2A. The tendency of the primer elongation characteristic for the particular BER substrate was similar in all extracts. The quantitative evaluation of product distribution according to their lengths is shown in Figure 2C (bottom row of diagrams). Taking into account the percentage of non-elongated primers and primers extended by one and several nucleotides, one can arrange the extracts according to the efficiency of DNA synthesis in the following order: HEK293T > Hgl > Mmu ≥3T3. It is worth noting that recombinant Polβ was the least efficient in the incorporation of the first nucleotide on the Flap substrate as compared to other BER substrates, while in HEK293T WCE comparable efficiency for all substrates was observed (Figure S2). This fact testifies to the direct involvement of the other cell extract proteins along with Polβ in DNA synthesis.

We intended to assess the influence of PAR synthesis on the efficiency of DNA synthesis in the presence of NAD^+^ in the extracts that differ significantly in the overall efficiency of PARylation. The data clearly demonstrate that the overall efficiency of DNA synthesis slightly increases in all cases irrespectively of the extract (compare Figure 2A with Figure 2B and in Figure 2C, top and bottom rows of diagrams). Generally, the effect of NAD^+^ was rather weak. In most cases, the amount of non-elongated initial primer decreased by less than 10% in the presence of NAD^+^ as compared with that in the absence of NAD^+^. However, the difference for the SP-BER substrate was slightly more pronounced in all extracts.

Interestingly, unusual products were formed in the presence of NAD^+^ in 3T3 and Mmu WCEs and in trace amounts in Hgl WCE. These products (marked in Figure 2B) were not registered in the absence of NAD^+^, and they were not characteristic for HEK293T WCE. We hypothesized that the unusual products appeared due to (ADP-ribosyl)ation of the primer during DNA synthesis. The possibility of PARP-dependent (ADP-ribosyl)ation of the terminal phosphate groups in DNA duplexes containing a gap/nick was demonstrated earlier [18–21], although under experimental conditions that did not involve DNA synthesis. The hypothesis of the primer (ADP-ribosyl)ation during DNA synthesis is confirmed by several experiments.

For convenience, mono(ADP-ribosyl)ation and poly(ADP-ribosyl)ation of an oligonucleotide to be elongated during the DNA synthesis is designated as MARylation and PARylation of a primer, respectively.

**Figure 3:**
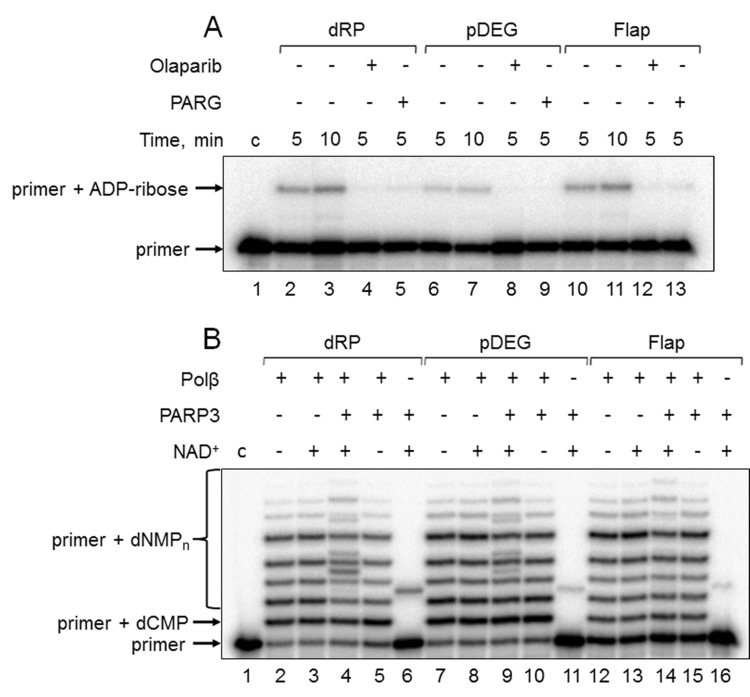
**Demonstration of primer MARylation in Mmu WCE (A) and in the system reconstituted from recombinant proteins (B).** (**A**) Mmu cell extract proteins (0.5 mg/mL) were incubated for 5 min with 100 nM DNA duplexes bearing dRP, pDEG, or flap in the presence of 5 mM MgCl_2_ and 0.5 mM NAD^+^ in the absence or presence of PARG and olaparib. (**B**) Recombinant proteins were incubated for 10 min with 100 nM DNA duplexes bearing dRP, pDEG, or flap in the presence of 5 mM MgCl_2,_ 0.1 mM dNTPs, and 0.5 mM NAD^+^ (when indicated). Lanes 1 in A and B correspond to the initial primer (control).

**Figure 4:**
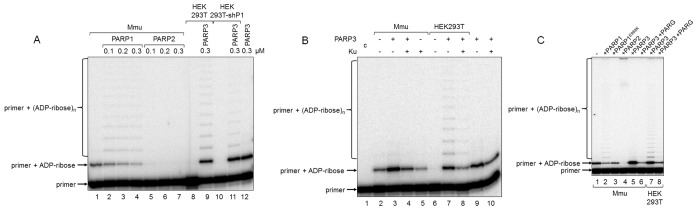
**Influence of exogenous proteins on (ADP-ribosyl)ation of primer in WCEs.** (**A**) WCE proteins in the absence or presence of extra recombinant proteins were incubated for 5 min with 100 nM DNA duplex dRP, 0.5 mM NAD^+^, and 5 mM spermine as described in the section ‘DNA (ADP-ribosyl)ation assay’. Recombinant PARP1, PARP2, or PARP3 were added to the extracts at the indicated concentrations prior to initiation of the (ADP-ribosyl)ation reaction. Lane 1, no extra recombinant proteins were added; lane 12, no extract proteins were added. (**B**) Mmu or HEK293T WCE proteins (0.5 mg/mL) were incubated for 5 min with 100 nM DNA duplex containing dRP moiety in the presence of 0.5 mM NAD^+^ and 5 mM spermine as described in the section ‘DNA (ADP-ribosyl)ation assay’. PARP3 and/or Ku (each at the final concentration of 300 nM) were added to the extracts prior to initiation of the (ADP-ribosyl)ation reaction. Lane 1 corresponds to the initial primer (control). (**C**) Mmu or HEK293T WCE proteins (0.5 mg/mL) were incubated at 37 °C for 5 min with 100 nM DNA duplex containing the dRP moiety in the presence of 0.5 mM NAD^+^ as described in the section ‘DNA (ADP-ribosyl)ation assay’. PARP1, PARP1^E988K^, PARP2, or PARP3 (the final concentrations of 300 nM) were initially added to the reaction mixtures and indicated. 50 nM PARG was added to some mixtures (lanes 6 and 8) after the reactions were stopped by the addition of EDTA, and the mixtures were incubated at 37 °C for another 10 min.

First, the products of (ADP-ribosyl)ation were not formed in the presence of olaparib, a PARP inhibitor, in the reaction mixtures with Mmu WCE (Figure 3A, compare lanes 2, 6, and 10 with lanes 4, 8, and 12). Second, the yield of the unusual products was significantly reduced in the presence of extra exogenous PARG (Figure 3A, compare lanes 2, 6, and 10 with lanes 5, 9, and 13), which is able to remove the ADP-ribose modification from DNA [21]. Third, the products of the same electrophoretic mobility appeared after incubation with mouse WCE and with mono(ADP-ribosyl)transferase PARP3 in the presence of NAD^+^ (Figure 4A, lanes 1 and 12, Figure 4B, lanes 2 and 9). Fourth, the indicated products are also formed in the reconstructed system containing Polβ and PARP3 instead of extract proteins (Figure 3B). Thus, our results together with the previously published data [19–21] allow for consideration of the unusual products as the MARylated primers containing the modifying group attached to the 5ʹ-terminal [^32^P]-phosphate group.

In this study, we used DNA substrates mimicking BER intermediates, which differ in the structure of the 5ʹ termini of downstream oligonucleotides. This fact appeared to influence the efficiency of primer MARylation (Figure 3B, compare lanes 6, 11, and 16). The efficiency of the upstream primer MARylation in the substrates decreases in a row: dRP > pDEG > Flap. DNAs bearing the 5ʹ-dRP and pDEG residues were not previously tested in the reaction of DNA (ADP-ribosyl)ation. MARylation of the upstream primer in an analogous flap-containing structure has already been demonstrated [19]. The efficiency of primer MARylation not only depends on the structure of the 5ʹ end of the downstream oligonucleotides but also differs for Mmu WCE and recombinant PARP3 (compare Figure 3A, lanes 3, 7, and 11 with Figure 3B, lanes 6, 11, and 16), which may reflect the processing of the groups at the 5ʹ termini of downstream oligonucleotides by the extract enzymes.

To determine how primer MARylation may interfere with the downstream stages of BER, we analyzed DNA synthesis in Mmu and Hgl WCEs in the presence or absence of ATP, NAD^+^, and olaparib (Figure S3 A and B). In the case of the reaction mixtures containing Mmu WCE and NAD^+^, the products that were ascribed to MARylated oligonucleotides fully or partially disappear in the presence of olaparib (Figure S3 A and B, lane 12 vs 13, lane 15 vs 16, and lane 18 vs 19) irrespectively of the presence of ATP. In all cases, the amount of the products that could be ascribed to the full-length chain insignificantly increases in the presence of ATP but their appearance is not related to DNA (ADP-ribosyl)ation.

We assumed that detectability of the MARylated primers would be dependent on the ratios of the PARP1, PARP2, PARP3, and PARG amounts, their activities in the extracts, and the relative affinities of these enzymes to damages in DNA. It is difficult to separately evaluate the activity of each PARP in the extracts and to compare the amounts of PARPs by immunological approaches due to different biological origins of the cells. However, significant information can be obtained in functional tests after the addition of certain purified proteins to the extracts.

**Figure 5:**
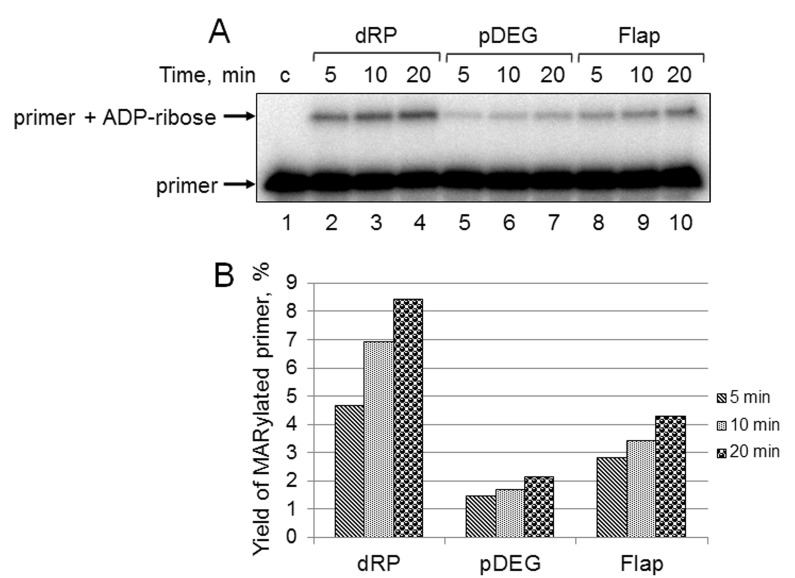
**Kinetics of primer MARylation in Mmu WCE (A) and quantification of the reaction products (B).** The Mmu cell extract proteins (0.5 mg/mL) were incubated for 5, 10, or 20 min with 100 nM DNA duplexes bearing dRP, pDEG, or flap in the presence of 0.5 mM NAD^+^ and 5 mM spermine as described in the section ‘DNA (ADP-ribosyl)ation assay’. Lane 1 corresponds to the initial primer (control). The yield of the MARylated primer (%) was calculated as the amount of the corresponding product normalized to overall DNA content in the lane.

First, we studied the kinetics of primer MARylation in the extracts using spermine as a cofactor for PARPs instead of magnesium ions to minimize primer degradation by endogenous nucleases in the WCE. The results for Mmu WCE are shown in Figure 5. In the absence of the DNA synthesis, MARylated primers can be consumed by either their further PARylation catalyzed by endogenous PARP1/PARP2 or the removal of the ADP-ribose residue by endogenous PARG. Slow time-dependent accumulation of the MARylated primer in Mmu WCE appears to reflect the balance between the activities of the above enzymes. The MARylated primers were registered in the extracts, which were characterized by low efficiency of PAR synthesis. This fact may indicate small amounts of PARP1 and PARP2 in these extracts. It should be noted that PARP1 contribution to the total PAR synthesis can reach 95% [26]. Indeed, Mmu WCE was the most inefficient in PAR synthesis compared to other extracts under study (Figure 1A). In addition, Mmu WCE demonstrates the lowest rate of PAR degradation (Figure 1B).

In the reconstituted system, both PARP1 and PARP2 were able to PARylate the upstream primer, which was preliminary MARylated, purified, and annealed to form a nick-containing DNA duplex, and a higher efficacy was observed for PARP2 [21]. Taking into account this result, we added extra PARP1, PARP2, or PARP3 into the extracts before PAR synthesis was initiated (Figure 4A). The addition of PARP1 to Mmu WCE led to concentration-dependent disappearance of the MARylated primer, which was accompanied by the appearance of the step-by-step elongation products (compare lane 1 with lanes 2–4 in Figure 4A), while the addition of PARP2 resulted in full disappearance of the MARylated primer even at the lowest concentration of PARP2 used (compare lane 1 with lanes 5–7 in Figure 4A). In addition, primer modification products with low electrophoretic mobility, i.e., with a higher PARylation level, can be observed close to the top of the gel (Figure 4C). The addition of extra PARP1 or PARP2 to Mmu extract enhances the amount of these products (Figure 4C, compare lanes 2 and 4 with lane 1), and the amount was higher for PARP2. PARP3, when added, does not influence the amount of low electrophoretic mobility products but only increases the amount of the MARylated primer (Figure 4C, lane 5 vs lane 1). Interestingly, PARP1^E988K^, the mutant form of PARP1, which has only mono(ADP-ribose) transferase activity, reduces the amount of the MARylated primer (Figure 4C, lane 3 vs lane 1). Taken together, these results may testify to a very rapid and efficient PARylation of the MARylated primer by extra PARP2. These results are corroborated by a considerably higher activity of PARP2 as compared to PARP1 in PARylation of a MARylated primer in a system reconstituted from recombinant proteins [21].

Using DNAs with unmodified or purified MARylated primers, it has been shown that PARylation of a MARylated primer can be performed quite efficiently in HEK293 WCE, while the products of *de novo* MARylation or PARylation of the unmodified primer were practically undetectable [21]. These data are in agreement with our results for HEK293T WCE (Figure 2B). This fact may be due to either a relatively low amount of PARP3 in human cells or a competition of DNA-binding cell proteins with PARP3 for DNA. Indeed, the addition of extra PARP3 to HEK293T WCE led to the appearance of the MARylated primer and products of its PARylation (Figure 4A, lane 9 vs lane 8 and lane 11 vs lane 10). Moreover, the pattern of the products resembles that obtained with Mmu WCE with extra PARP1 (Figure 4A, lanes 2–4). A lower amount of the PARylated primer is detected in the extract of HEK293T cells, in which PARP1 production is partially inhibited by expression of a specific shRNA (HEK293T-shP1), (Figure 4A, lane 11 vs lane 9). The efficiency of conversion of the MARylated primer, which was synthesized in the WCEs of HEK293T-shP1 and HEK293T in the presence of extra PARP3, to the PARylated primer was 5.4% and 24.9%, respectively (compare lanes 9 and 11 in Figure 4A). It should be emphasized that the yield of PAR in WCEs of HEK293T-shP1 as compared to HEK293T was about 30% as determined by the test on the paper filters (data not shown).

Another cell protein that may interfere with MARylation of primers by PARP3 at the 5ʹ ends is Ku antigen (Ku), which is composed of the Ku80 and Ku70 polypeptide chains named according to their molecular masses of about 80 and 70 kDa. Ku binds double-stranded DNA ends with high affinity and is well-known for its central role as a DNA end-binding factor at the initial stage of the classical nonhomologous end-joining pathway, which is the main DNA double-strand break (DSB) repair pathway in mammalian cells [29–31]. The data on the interference of extra Ku with DNA (ADP-ribosyl)ation in the extracts are shown in Figure 4B. The addition of Ku to Mmu WCE reduced the level of primer MARylation by endogenous PARP3 by 40% (lane 5 vs lane 2). The inhibitory effect of Ku was also manifested when both PARP3 and Ku were added to the extract (compare lanes 3 and 4) or in the reaction mixtures containing purified PARP3 and Ku (compare lanes 9 and 10). In HEK293T WCE, the addition of PARP3, as expected, resulted in the appearance of the MARylated primer (compare lanes 6 and 7) and extra Ku interfered with this process (compare lanes 7 and 8).

### Affinity labeling of cell extract proteins by chemically reactive DNAs mimicking BER intermediates

**Figure 6:**
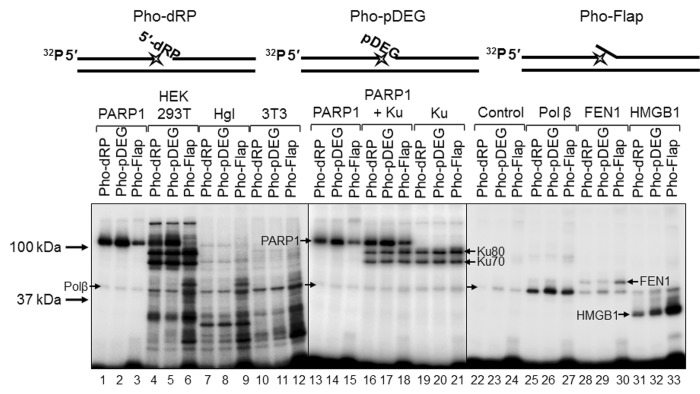
**Interaction of proteins with different types of photoreactive DNA.** Photoaffinity modification was performed as described in the section ‘Photoaffinity modification of proteins’ using 100 nM DNAs and 1 mg/mL cell extract proteins (HEK293T, lanes 4–6; Hgl, lanes 7–9; 3T3, lanes 10–12), as well as purified PARP1 (100 nM, lanes 1–3 and 13–15), PARP1 + Ku (100 nM each, lanes 16–18), Ku (100 nM, lanes 19–21), Polβ (200 nM, lanes 25–27), FEN1 (100 nM, lanes 28–30), and HMGB1 (300 nM, lanes 31–33). Lanes 22–24 (control) correspond to the UV-light irradiated aliquots of the reaction mixtures for photoreactive DNA synthesis, which contained 100 nM Polβ. The proteins were separated by 12.5% SDS-PAGE and the proteins cross-linked to [^32^P]-labeled DNAs were visualized by autoradiography. The structures of the photoreactive DNAs are schematically shown at the top. The asterisk denotes the FAP-dCMP residue.

For a better understanding of the mechanisms and the role of DNA (ADP-ribosyl)ation, especially taking into account the above data, it is important to obtain information about the amounts in the extracts of the target proteins and other DNA binding, which may influence the binding of target proteins with DNA. Comparison of the amount of some proteins in the cell extracts may be performed by using DNA, which mimics the intermediates of the corresponding DNA repair process and contains photoactivatable groups. Synthesis of the photoreactive BER substrates can be carried out by the attachment of dCMP analogs to the 3ʹ end of a radioactively labeled primer using recombinant Polβ [32]. Here, we use exo-N-{2-[N-(4-azido-2,5-difluoro-3-chloropyridine-6-yl)-3-aminopropionyl]amino-ethyl}-2′-deoxycitidine-5′-triphosphate (FAP-dCTP) as a substrate. The chosen conditions for the synthesis of photoreactive DNA provided a full elongation of the primer by FAP-dCMP in all three BER substrates (Figure S4). The DNA synthesis was stopped by the addition of EDTA, and the reaction mixture was further used as a source of the photoreactive DNAs. It should be noted that EDTA is a mandatory component of the reaction mixtures, which prevents DNA hydrolysis s by endogenous nucleases in the extracts. The WCE or recombinant proteins were incubated with photoreactive DNAs for binding, followed by UV-light irradiation for DNA-protein cross-linking. The data on the cross-linking of proteins to the photoreactive DNAs are shown in Figure 6. The main difference between the patterns of protein cross-linking consists in extremely intensive labeling of high molecular weight proteins (70–120 kDa) in HEK293T WCE (Figure 6, lanes 4–6 vs lanes 7–9 and 10–12). Taking into account the apparent molecular masses of the observed products and our previous data concerning the photoaffinity labeling of proteins in the extracts of mammalian cells [15,33,34], we can assume that these three highly intensive bands correspond to the cross-linking products formed by PARP1, Ku80, and Ku70 (from top to bottom). The cross-linking of DNAs to individual proteins or their combinations (Figure 6, lanes 4–6 vs lanes 1–3 and lanes 13–21) led to the appearance of bands with corresponding mobility thus confirming our hypothesis. Interestingly, the intensity of the bands attributed to PARP1 cross-linking products varied depending on the type of photoreactive DNA, and the lowest intensity was observed for Pho-Flap (Figure 6, lanes 1, 2 vs lane 3; lanes 13, 14 vs lane 15). The analogous tendency is typical of HEK293T WCE (Figure 6, lanes 4, 5 vs lane 6). Only low-intensity bands, which can be attributed to the products of PARP1 cross-linking were detected in Hgl WCE, (Figure 6, lanes 7–9 vs lanes 1–3), while the corresponding products were not practically observed in 3T3 WCE (Figure 6, lanes 10–12). This observation correlates with the data shown in Figure 1A concerning an extremely high difference in the total PARylation efficiency between human and rodent cell extracts. In HEK293T WCE, the bands corresponding to the products of Ku protein cross-linking are very abundant (Figure 6, lanes 4–6), while no products of cross-linking, which could be unambiguously attributed to Ku, were observed in rodent cell extracts. Importantly, very intensive bands corresponding to the products of the Ku protein labeling by an analogous photoreactive DNA were observed in HeLa WCE [34] but not in the mouse embryonic fibroblast extract [15]. This is fully consistent with previously published data on a higher abundance of Ku in cells of primates compared to other mammals [35].

The intensity of the bands corresponding to the cross-linking products with molecular mass of about 50 kDa is comparable for all extracts, and these products may be easily attributed to recombinant Polβ since the preparations of photoreactive DNA already contain this protein, which was used for the incorporation of FAP-dCMP (Figure 6, lanes 22–24). Therefore, the product corresponding to Polβ is present in all samples and can serve as an internal control. It should be noted that 200 nM Polβ (lanes 25–27) produced stronger signal than endogenous Polβ of the extracts. The intensive products with a higher apparent molecular mass than that of Polβ are observed in lanes 6, 9, and 12 corresponding to the Pho-Flap DNA. These products can be attributed to FEN1. Purified recombinant FEN1 (lanes 28–30) generates the products with the expected electrophoretic mobility.

Another DNA-binding protein, which may be involved in the BER process, is HMGB1. HMGB1 is a nuclear nonhistone DNA-binding protein, which belongs to the high-mobility group box family of proteins [36]. We have shown earlier that HMGB1 is a BER cofactor capable of modulating the BER capacity in cells [37]. Hgl and 3T3 WCEs demonstrate comparable levels of the products, which can be attributed to HMGB1 (lanes 7–12) according to their electrophoretic mobility corresponding to the mobility of the products formed by purified HMGB1 (lanes 31–33). In the case of HEK293T WCE, the corresponding bands have higher intensities (lanes 4–6 vs lanes 7–12). In all cases, the Pho-Flap DNA more efficiently cross-links to HMGB1 than other photoreactive DNAs (compare lanes 6, 9, 12, and 33 with lanes 4, 5, 6, 7, 8, 10, 11, 31, and 32). This pattern of cross-linking is in line with the ability of HMGB1 to bind more efficiently flap-containing DNAs than other BER substrates as revealed by EMSA [37].

Using Pho-dRP DNA, we performed photoaffinity modification of all three recombinant PARPs presented alone or in different combinations and also 3T3, Hgl, and HEK293T cell extracts (Figure S5). PARP3 demonstrated the considerably lower level of cross-linking as compared to PARP1 and PARP2 (lane 3 vs lanes 1 and 2) at the equal concentrations of DNA and proteins. No products, which could be ascribed to cross-links of PARP2 with DNA according to electrophoretic mobility, were detected in the extracts (compare lane 8 with lanes 9–11); this fact may indicate a small number of PARP2 copies. The nature of DNA-protein covalent adducts in HEK293T and Hgl WCEs, which we attribute to PARP1, was additionally confirmed by cross-linking in the presence of NAD^+^ (Figure S5B). The amount of the PARP1-dependent protein–DNA complex was reduced and slower migrating material was now observed both with purified PARP1 (compare lanes 1–3 and lanes 13–15 with lanes 4–6 and lanes 16–18 in Figure 5S B) and in WCEs (compare lanes 7–9 with lanes 10–12 and lanes 19–21 with lanes 22–24 in Figure 5S B). This fact is consistent with cross-linking of DNA probes with PARylated PARP1. The smeared slower migrating material appeared due to variable length of the attached PAR. This phenomenon has already been demonstrated by us earlier [6,15].

## DISCUSSION

Earlier we have compared the activity of the several enzymes of the BER system and PARylation of the extract proteins in the extracts of naked mole rat and mouse cells [1] but the effect of the PAR synthesis/degradation on DNA synthesis was not addressed. Here we studied DNA synthesis and the effect of PAR synthesis on this process in more detail, having added in the study an extract of human cells, another long-lived mammal.

It should be noted that the higher effective PAR synthesis in Hgl and human WCEs as compared to that in mouse WCEs is fully consistent with the previously published data on the higher efficiency of this process in the cells of long-lived organisms [1,27,28].

The patterns of DNA synthesis products were quite similar in all extracts, and the higher efficiency of the DNA synthesis was observed in human and Hgl cell extracts in both the gap-filling step and the further primer elongation. In the presence of NAD^+^, DNA synthesis on all substrates became more efficient with a tendency of a higher efficiency on the short-patch substrate. Interestingly, the changes occurred almost to the same extent in all extracts irrespectively of both the total efficiency of PAR synthesis (Figure 1) and the amount of PARP1, which was evaluated by cross-linking of the extract proteins with the photoreactive BER substrates (Figure 6, lanes 4–12). Earlier, using a system reconstituted from purified proteins and bovine testis nuclear extract supplemented with recombinant PARP1 we have revealed that PARP1 may act as a regulator of Polβ activity in the LP-BER pathway and that PARylation of PARP1 plays a crucial role in ensuring of the Polβ-mediated DNA synthesis [17,40]. It is important to note that we used in those works a 5–10-fold excess of PARP1 over DNA and the BER substrates were represented by DNA duplexes with a single-nucleotide gap flanked at the 5ʹ end by either phosphate (the SP pathway substrate) or 3-hydroxy-2-hydroxymethyltetrahydrofuran with phosphate (the LP pathway substrate). It should be taken into account that PARP1 is not the only protein factor interfering with the activity of Polβ on the BER substrates. It was also revealed that APE1, FEN1, and HMGB1 can influence the efficiency of DNA synthesis in BER by different protein-protein and DNA-protein interactions [17,37–41]. The overall effect depends on several factors, i.e., the type of DNA duplex, stoichiometry of interacting biopolymers, and the presence of other DNA-binding proteins.

In general, the influence of NAD^+^ on the efficiency of DNA synthesis in our system is not high in amplitude. However, we revealed a new phenomenon, i.e., PARylation of DNA substrates during DNA repair synthesis. The details of DNA (ADP-ribosyl)ation by PARP1, PARP2, and PARP3 have been already studied in the works of our laboratory and other research groups [18–21]. However, the association between DNA (ADP-ribosyl)ation and DNA synthesis has not been previously considered. Here, we demonstrated that PARP3 was able to MARylate primers during DNA synthesis catalyzed by recombinant Polβ and DNA polymerase(s) in whole-cell extracts on the model BER substrates bearing the 5ʹ-terminal phosphate group at blunt double-stranded ends. These short DNA structures with double-stranded ends are not typical BER substrates. However, they can appear during exposure to some genotoxic agents, such as ionizing radiation. Some cellular proteins, which are able to efficiently bind DSB ends, may interfere with MARylation at the 5ʹ-terminal phosphate group. The Ku protein is known to efficiently bind DSB ends during nonhomologous end joining to protect the broken DNA and to recruit the downstream repair factors [42]. This protein inhibits MARylation of the primer when added to Mmu WCE even in the presence of extra PARP3 (Figure 4B). MARylation of the primer was not detected in HEK293T WCE. This fact may be related to a weak PARP3 activity because of a low content or its competition with Ku, whose concentration is considerably higher in this extract than in other extracts, as was determined by photocross-linking of DNA. Given the high content of PARP1 in HEK293T WCE, it cannot be ruled out that PARP1 is also able to compete with PARP3 for DNA although this was not directly confirmed in the current work.

It should be emphasized that a reliable comparison of the protein amounts in the extracts is not a trivial task if the cells derived from different species. The Western blot analysis is reagent- and time-consuming process and has a limitation because requires the purified target proteins for calibration. The amount of mRNA encoding a protein does not always unambiguously reflect the level of protein expression/activity [43]. As an alternative way for comparison of the amount of the particular proteins in the cell extracts affinity modification may be used. The DNA-protein cross-linking pattern visualizes a set of proteins of a particular extract that react with a specific DNA. Moreover, when in the extracts the amount of target proteins were evaluated by the yield of the DNA-protein cross-links and immunochemical approaches, the positive correlation has been demonstrated [34].

The additional way for regulation of growth of the DNA-linked PAR chain can be realized via protein-protein interactions of PARP1/2 with the specific modulation factors. HPF1 appears to be the most suitable. HPF1, the PARP1-accessory factor, as has been recently shown [44–46], is able to alter the profile of the PARylated proteins in mammalian cell extracts. Moreover, HPF1 alters the specificity of both PARP1and PARP2, but not PARP3, and confers serine specificity as a target amino acid for the PAR attachment. There are additional examples of modulation protein factors. The Y-box-binding protein 1 physically interacts with PARP1 and other BER proteins and affects the PARP1 activity [47,48]. The Timeless protein specifically binds to the catalytic region of PARP1, but not other PARP family members, to promote homologous recombination repair [49,50]. Thus, one cannot exclude that the variety of specific protein partners could alter the activity/specificity of PARPs towards particular targets including DNA.

The further fate of the ADP-ribose attached to the 5ʹ-terminal phosphate group of a primer appears to depend on the PARP1, PARP2, and PARG ratios. The ADP-ribose residue can be removed by PARG as shown in this study and previously [19–21]. Alternatively, the ADP-ribose residue can serve as a primary substrate for the growth of a PAR chain catalyzed by PARP1 and/or PARP2. The addition of PARP1 to Mmu WCE resulted in the products bearing several ADP-ribose residues with simultaneous consumption of the MARylated primer, while the addition of PARP2 led to the almost complete disappearance of the MARylated primer along with the emergence of low electrophoretic mobility products corresponding to the PARylated primer (Figure 4C). The observed patterns of primer PARylation are in full agreement with the specificity of PARP1 and PARP2 action on an isolated MARylated primer [21]. As shown, PARP2 displays a higher activity and processivity than PARP1 in DNA PARylation. PARP1 and PARP2 themselves demonstrated a low activity in PARylation of a primer phosphorylated at the 5ʹ end as compared to a preliminary MARylated primer.

The data on the participation of PARP3 in DSB repair are based on several evidences, with the effect of PARP3 on the regulation of DNA resection playing a crucial role [12–14]. Limitations of DSB ends resection can be achieved by not only recruitment of the specific proteins, but also a modification of DNA ends. The specificity of DNA modification catalyzed by PARP3, i.e., more efficient attachment of the ADP-ribose residue to the 5ʹ-terminal phosphate DSB ends compared to that in the nick or gap [19–21], supposes the PARP3-mediated protection. The reversibility of the PAR/MAR DNA modification by PARG and other PAR/MAR processing proteins is in line with the temporal protection hypothesis [19–21].

The PARP3-mediated MARylation at the 5ʹ-phosphorylated DSB termini is considerably more efficient in DNA substrates containing nicks/gaps compared to that in double-stranded DNA without these damages [19,20]. Taking into account this fact, one can assume that this protective mechanism may be relevant when DSB and the nick/gap are located within one-two turns of the DNA helix providing a lag to repair a single-strand break.

PARP1 and PARP2 may also play a role in the regulation of DSB ends resection by either direct PARylation of the ends or PAR synthesis utilizing DNA MARylated by PARP3. It should be noted that the activities of PARPs in DNA modification are strongly dependent on the DNA structure (full or partial DNA duplex, the presence and positions of nicks/gaps, the presence of the phosphate group at the 5ʹ or 3ʹ end), and the specificity depends on the type of PARP [18–21].

It is worthy of note that MARylation of the 5ʹ-terminal phosphate at DSBs facilitates DNA PARylation by PARP1 and PARP2 as compared to the same DNA bearing unmodified 5ʹ-phosphate [21], thus indicating a particular role of PARP3. Interestingly, no significant difference in the yield of the PARylated primer with high molecular weight was detected in spite of an extremely high content of PARP1 in HEK293T WCE compared to other cell extracts. This observation also emphasizes the crucial role of PARP3 in DNA PARylation in cells. Enhanced expression of PARP3 during the etoposide treatment, which inhibits topoisomerase II, thereby inducing DSBs, was found in MDA-MB231 cells [11]. This fact is in line with possible involvement of DNA PARylation in the regulation of DBS repair. The PAR/MAR dependent influence on the resection of DSB ends may be essential for the cells with a low content of the Ku protein.

In general, in all studied extracts the difference in the efficiency of DNA repair synthesis on the BER substrates is moderate with a tendency of higher activity in the cells of long-lived organisms, naked mole rat and human. At the same time, in mouse WCEs, which are characterized by lower level of the total PAR synthesis and lower efficiency of the primer elongation the functional specificity of the MARylation/PARylation system is clearly detected, which was manifested in MARylation of DNA ends. Data obtained with extra proteins suggest that the real ratios of PARPs and effect of some DNA-binding proteins in the extracts may even change the targets of PARylation to influence the regulation of DNA repair by additional way.

## MATERIALS AND METHODS

### Materials

**[**γ-^32^P]ATP (5000 Ci/mmol) and [α-^32^P]ATP (3000 Ci/mmol) were produced in the Laboratory of Biotechnology, ICBFM SB RAS. *Exo-N*-{2-[*N*-(4-azido-2,5-difluoro-3-chloropyridine-6-yl)-3-aminopropionyl]aminoethyl}-2′-deoxycitidine-5′-triphosphate (FAP-dCTP) was synthesized as described in [32]. Recombinant T4 polynucleotide kinase and *E. coli* uracil-DNA glycosylase (Ung) were from Biosan (Russia). Plasmids bearing cDNA of human Polβ and flap endonuclease 1 (FEN1) were kindly provided by Prof. S.H. Wilson (NIEHS, NIH, USA). Recombinant proteins Polβ and FEN1 were overexpressed in *E. coli* and purified as described previously [51,52]. HMGB1 was purified from calf thymus as described in [53] with additional purification on Q-Sepharose. Vectors encoding human PARP1, murine PARP2, and murine PARG were kindly provided by Dr. V. Schreiber (ESBS, Illkirch, France). Plasmid encoding human PARP3 was kindly provided by Dr. A. Ishchenko (Gustave Roussy, Université Paris-Saclay, France). Recombinant PARP1 and PARP2 were purified as described in [54]. PARP3 was purified according to [21]. Purification of PARG was performed as in [55]. PARP1^E988K^ was kindly provided by K. Naumenko (ICBFM SB RAS). Human Ku protein was purified from HeLa cell extract by ammonium sulfate fractionation (45–65% of saturation), followed by successive chromatography procedures on DEAE Support (Bio-Rad, USA), Q-Sepharose (GE Healthcare, USA), and ds-DNA-Cellulose (ICN, USA). HEK293T and HEK293T-shP1 cells kindly provided by Prof. O. Dontsova (Moscow State University, Russia) were cultured in the DMEM medium (Biolot, Russia) in the presence of 10% FBS (HyClone, USA), 100 unit/mL penicillin (Invitrogen, USA) and 100 µg/mL streptomycin (Invitrogen, USA) with 5% CO_2_ in a humidified atmosphere. *Mus musculus* primary fibroblasts (Mmu) were cultured in alpha MEM (Gibco) supplemented with 15% FBS (Gibco), penicillin, streptomycin, and amphotericin B at 37 °C in 5% CO_2_. Mouse fibroblasts 3T3 were cultured in high glucose DMEM supplemented with 10% FBS (Biosera), penicillin, and streptomycin at 37 °C in 5% CO_2_. *H. glaber* cells were cultured in alpha MEM (Gibco) supplemented with 15% FBS (Gibco), 10% AmnioMAX II Complete Medium (Gibco), 5 ng/ml bFGF, 10^5^ U/L penicillin, and 100 mg/L streptomycin, 2.5 mg/L amphotericin B at 32 °C in 5% CO_2_. After the cells reached confluence, the culture flasks were washed with PBS, and the cells were treated with the trypsin/Versene (1:1) solution (HyClone, USA), collected by centrifugation, and washed with PBS, followed by additional centrifugation. Cell extracts were prepared as described in [56]. The reagents for electrophoresis and buffer components were from Sigma (USA).

### Oligonucleotides

Oligodeoxyribonucleotides were synthesized in the Laboratory of Medicinal Chemistry, ICBFM SB RAS (see Table 1). The upstream oligonucleotide (Up) was 5′-[^32^P]-labeled with T4 polynucleotide kinase and [γ-^32^P]ATP according to [57]. Unreacted [γ-^32^P]ATP was removed using a MicroSpin G-25 column (GE Healthcare, USA) according to the manufacturer’s protocol.

**Table 1:** Oligonucleotide sequences used in this study.

Name	Sequence
Up 1	5’- GGGAGGCCCTGGCGTT-3’
Down U 1	5’- p**U**CCCGGCTTAGTCGCC-3’
Down DEG 1	5’- p-**DEG**-CCCGGCTTAGTCGCC-3’
Down Flap 1	5’- **GATAAC**CCCGGCTTAGTCGCC-3’
Template 1	5’- GGCGACTAAGCCGGGGAACGCCAGGGCCTCCC-3’
Up 2	5’-GGCGACTAAGCCGGG-3’
Down U 2	5’-p**U**AACGCCAGGGCCTCCC-3’
Down DEG 2	5’-p-**DEG-**AACGCCAGGGCCTCCC-3’
Down Flap 2	5’-**GATAAC**AACGCCAGGGCCTCCC-3’
Template 2	5’-GGGAGGCCCTGGCGTT**G**CCCGGCTTAGTCGCC-3’

DNA duplexes were obtained by annealing of the upstream (Up), downstream (Down U/DEG/Flap), and template (complementary to Up and Down) oligonucleotides in the 1:2:2 ratio by heating a solution at 97 °C for 5 min, followed by slow cooling to room temperature. The uracil residue in the Down U oligonucleotide was removed immediately before experiments by the Ung treatment (1 U/µL) at 37 °C for 30 min to generate the 5′-dRP residue. DNA duplexes prepared from oligonucleotides “1” were used in most of the experiments except photoaffinity labeling of proteins. The analogous system of BER substrates prepared from oligonucleotides “2” was used for the synthesis of photoreactive DNA since the sequence of Template 2 provides incorporation of one dCMP or FAP-dCMP in contrast to system 1, which allowed for incorporation of four dCMP residues in a row.

### DNA synthesis

The standard reaction mixtures (10 µL) contained 50 mM Tris-HCl (pH 8.0), 40 mM NaCl, 1 mM DTT, 0.1 mg/mL BSA, 5 mM MgCl_2_, 0.1 mM dNTPs, 100 nM 5′-[^32^P]-labeled DNA substrates, and 0.5 mg/mL cell extract proteins or 10 nM Polβ; 0.5 mM NAD^+^ and/or 1 mM ATP were added when indicated. Prior to DNA synthesis, DNA substrates were treated by Ung as described above to obtain the 5ʹ-dRP residues. After adding cell extract proteins or Polβ, the reaction mixtures were incubated at 37 °C for 5 or 10 min as indicated in the legends of the figures. The reactions were stopped by the addition of EDTA at the final concentration of 20 mM, followed by incubation at 0 °C. The products were analyzed by electrophoresis in 20% polyacrylamide gel containing 7 M urea [57]. The gels were dried and subjected to phosphorimaging for quantification on a Biomolecular Imager Typhoon FLA 9500 (GE Healthcare Life Sciences, USA) and the Quantity One software (Bio-Rad, USA).

### DNA (ADP-ribosyl)ation assay

The standard reaction mixtures (10 µL) contained 50 mM Tris-HCl (pH 8.0), 40 mM NaCl, 1 mM DTT, 0.1 mg/mL BSA, 5 mM spermine (or 5 mM MgCl_2_ where indicated), 0.5 mM NAD^+^, 100 nM 5′-[^32^P]-labeled DNA substrate, and 0.5 mg/mL cell extract proteins and/or purified proteins at indicated concentrations. In addition, the reaction mixtures were supplemented with 10 µM olaparib, which was preliminarily dissolved in DMSO in the case of PARP inhibition or 10% DMSO in other cases. Prior to DNA (ADP-ribosyl)ation, the DNA substrates were treated by Ung as described above to obtain the dRP residues. After adding the proteins, the reaction mixtures were incubated at 37 °C for the time specified in the legends. The reactions were terminated by the addition of 10 µM olaparib, and the reaction mixtures were placed in an ice bath and analyzed as described in the previous section.

### Photoreactive DNAs

Photoreactive DNAs were synthesized using purified Polβ and FAP-dCTP as a substrate. We used nick-containing DNA duplexes with the upstream primer ^32^P-labeled at the 5′-end. The downstream primer contained either the dRP, or pDEG, or 6-nt flap at its 5′-end. In this case, DNA duplexes were prepared from oligonucleotides Up 2, Down U 2, Down DEG 2, Down Flap 2, and Template 2 (Table 1) with the template sequence, which made it possible to incorporate one dCMP or FAP-dCMP in contrast to system 1, in which four dCMP residues in a row can be incorporated.

The standard reaction mixture for the synthesis of photoreactive DNA contained 50 mM Tris-HCl (pH 8.0), 40 mM NaCl, 1 mM DTT, 0.1 mg/mL BSA, 5 mM MgCl_2_, 100 nM 5′-[^32^P]-labeled DNA substrate, 50 µM FAP-dCTP, and 100 nM Polβ. The reaction mixtures were incubated at 37 °C for 30 min, followed by the addition of 20 mM EDTA to bind Mg^2+^ ions, that inhibits the Polβ activity. This reaction mixture was used as a source of photoreactive DNA without additional purification.

### Photoaffinity modification of proteins

The reaction mixtures for UV-inducible cross-linking (10 µL) contained 50 mM Tris-HCl (pH 8.0), 65 mM NaCl, 1 mM DTT, 0.1 mg/mL BSA, 15 mM EDTA, 100 nM photoreactive DNA, and 1 mg/mL cell extract proteins or 100 (300) nM purified proteins as indicated in the legends. The reaction mixtures were assembled on ice. Photolysis was induced by UV light using a Bio-Link-BLX cross-linker (VILBER-LOURMAT) at 312 nm, 1.5 J/cm^2^ for 5 min. After irradiation, the reaction mixtures were supplemented with Laemmli sample buffer and heated for 5 min at 97 °C. The products were analyzed by SDS-PAG electrophoresis in 12.5% gel as described in [58], followed by autoradiography using a Biomolecular Imager Typhoon FLA 9500 (GE Healthcare Life Sciences, USA) and the Quantity One software (Bio-Rad, USA).

### [^32^P]-NAD^+^ synthesis

The synthesis of radioactive NAD^+^ was carried out from [α-^32^P]-ATP according to [59] with modifications described in [9]. The reaction mixtures containing 1 mM ATP, 10 MBq of [α-^32^P]ATP, 20 mM MgCl_2_, 2 mM β-nicotinamide mononucleotide, and 5 mg/mL nicotinamide nucleotide adenylyltransferase in 25 mM Tris-HCl (pH 7.5) were incubated at 37 °C for 60 min and stopped by heating to 90 °C for 3 min. After removal of a denatured protein by centrifugation, the solution was used as a source of NAD^+^ without purification.

### Synthesis and degradation of PAR in WCEs

### *PARP activity assay*

The reaction mixtures (10 μL) containing 50 mM Tris-HCl (pH 8.0), 40 mM NaCl, 8 mM MgCl_2_, 1 mM DTT, 0.1 mg/mL BSA, 0.6 A_260_/mL of activated DNA or 0.1 μM the SP-BER substrate, and 20 μM [^32^P]-NAD^+^ were assembled on ice. The cell extract proteins at the final concentration of 0.5 or 1.0 mg/mL or 10 nM PARP1 were added as indicated in the figure legends. The reaction mixtures were incubated at 37 °C for 1 min and the reaction was stopped by the addition of Laemmli sample buffer [58]. The mixtures were heated at 97 °C for 10 min and the products were further analyzed by 12.5% SDS-PAG electrophoresis [58], followed by autoradiography. Alternatively, the reaction mixtures (50 μL) of the same composition were incubated at 37 °C, and aliquots (5 μL) were taken at certain intervals. The reaction was stopped by dropping the aliquot on the Whatman 1 paper filters pre-impregnated with trichloroacetic acid (TCA). PAR attached to proteins was precipitated on the filters in the presence of TCA. To remove unreacted NAD^+^, the dried filters were washed three times with 150 mL of 5% ice-cold TCA, the rest of TCA was removed from paper by 90% ethanol, and filters were dried and subjected to autoradiography for quantification using a Biomolecular Imager Typhoon FLA 9500 (GE Healthcare Life Sciences, USA) and the Quantity One software (Bio-Rad, USA).

### *PAR degradation ass*

[^32^P]-PAR was synthesized using recombinant 10 nM PARP1 in the reaction mixture (190 μL) containing 50 mM Tris-HCl (pH 8.0), 40 mM NaCl, 8 mM MgCl_2_, 1 mM DTT, 0.1 mg/ml BSA, 0.6 A_260_/mL of activated DNA, and 20 μM [^32^P]-NAD^+^ for 10 min at 37 °C, then the reaction of PAR synthesis was stopped by EDTA at the final concentration of 20 mM. The control aliquot of 5 μL was withdrawn and dropped to the Whatman 1 paper. The rest of the reaction mixture was used as a source of PAR. One of the cell extracts or recombinant PARG (all in volumes of 2.5 μL) was added to the aliquots (35 μL) of this reaction mixture to the final concentration of 0.5 mg/mL for the extract proteins and of 10 nM for PARG. The reaction mixtures were incubated at 37 °C, and aliquots (5 μL) taken at certain intervals were loaded onto paper filters and treated as described in the previous section.

## Supplementary Material

Supplementary Figures
