# Supplementary material for: Poly(ADP-ribosyl)ation and DNA repair synthesis in the extracts of naked mole rat, mouse, and human cells
Source: Aging (Albany NY). 2019 May 13;11(9):2852–73. doi: 10.18632/aging.101959 (PMC6535076; doi:10.18632/aging.101959)
Supplement: Supplementary Figures [file aging-11-101959-s001.pdf]

SUPPLEMENTARY FIGURES

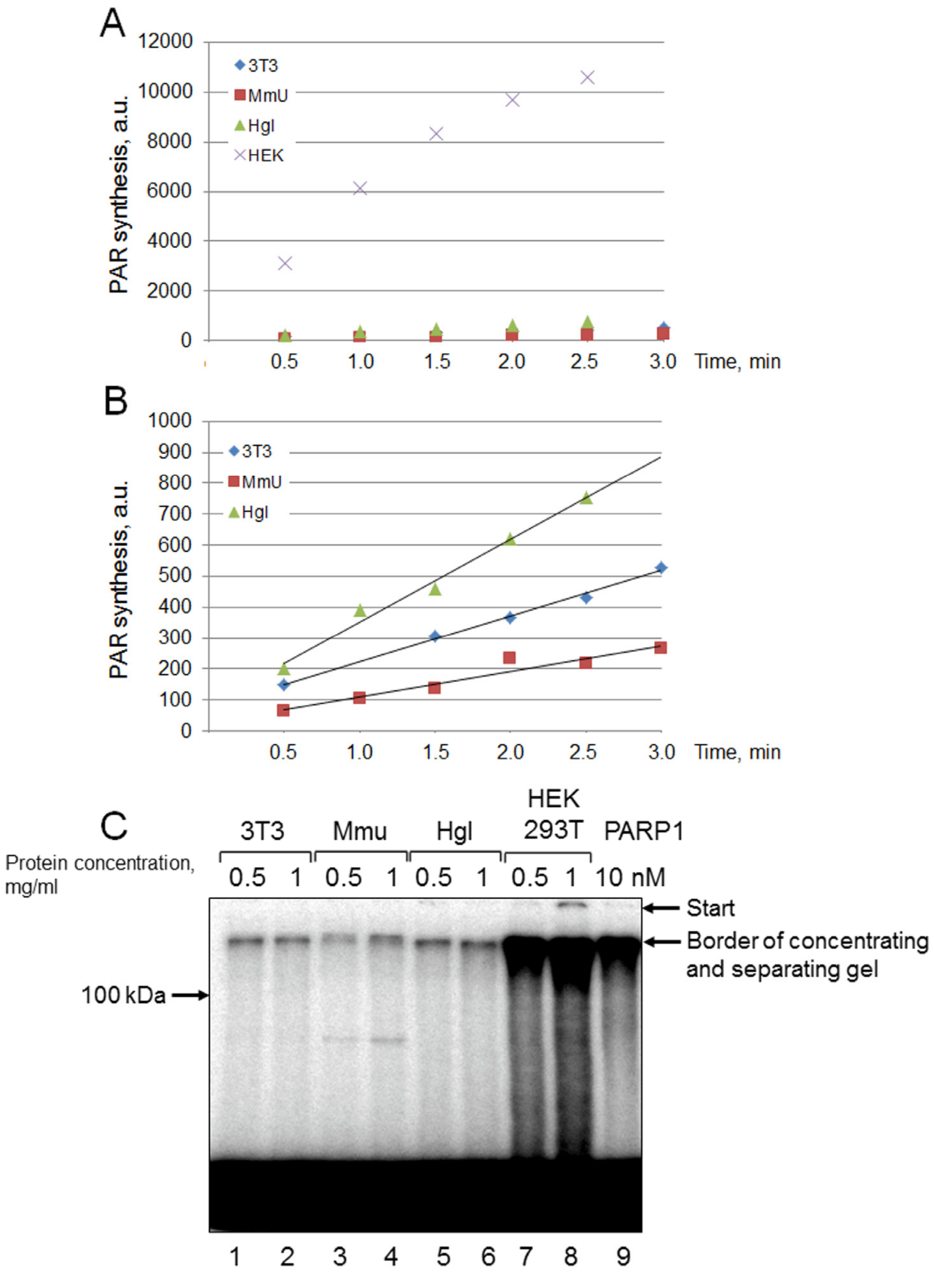

Figure S1. Efficiency of PAR synthesis in the cell extracts. (**A** and **B**) Kinetic curves of PAR synthesis as determined by adsorption of PAR on paper (see section 'Synthesis and degradation of PAR in the extracts'). The level of PAR synthesis is determined in arbitrary phosphorimaging units. B - same as in A for the rodent cell extracts, but in another scale. (**C**) Electrophoretic analysis of PAR synthesized for 1 min by endogenous PARPs in cell extracts at two protein concentrations (0.5 and 1.0 mg/ml) or by 10 nM recombinant human PARP1 (autoradiograph of the gel). The cell lines are indicated.

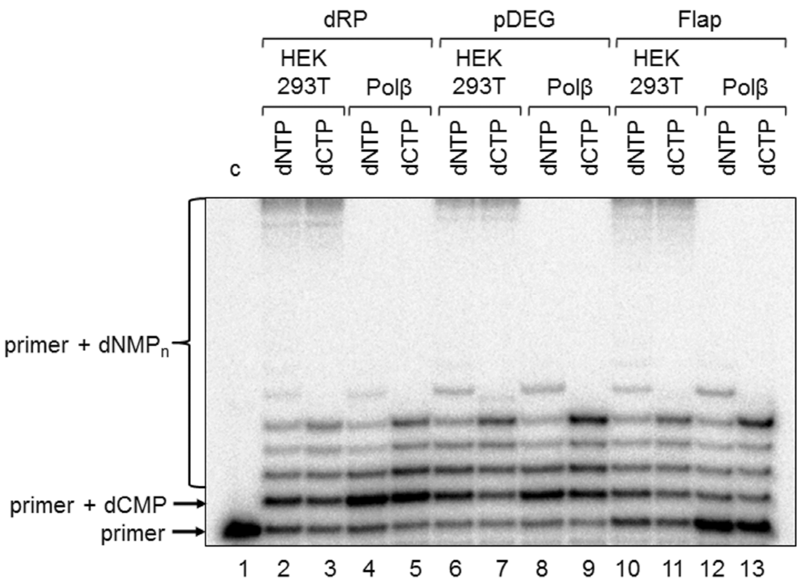

Figure S2. DNA synthesis by HEK293T cell extract proteins and Polβ on different DNA substrates. The reaction mixtures containing 100 nM 5′-[^{32}P]-labeled DNA substrate, 0.1 mM dNTPs or 0.1 mM dCTP, and 0.5 mg/ml cell extract proteins or 10 nM Polβ were incubated at 37 °C for 10 min. The types of DNA substrates are indicated.

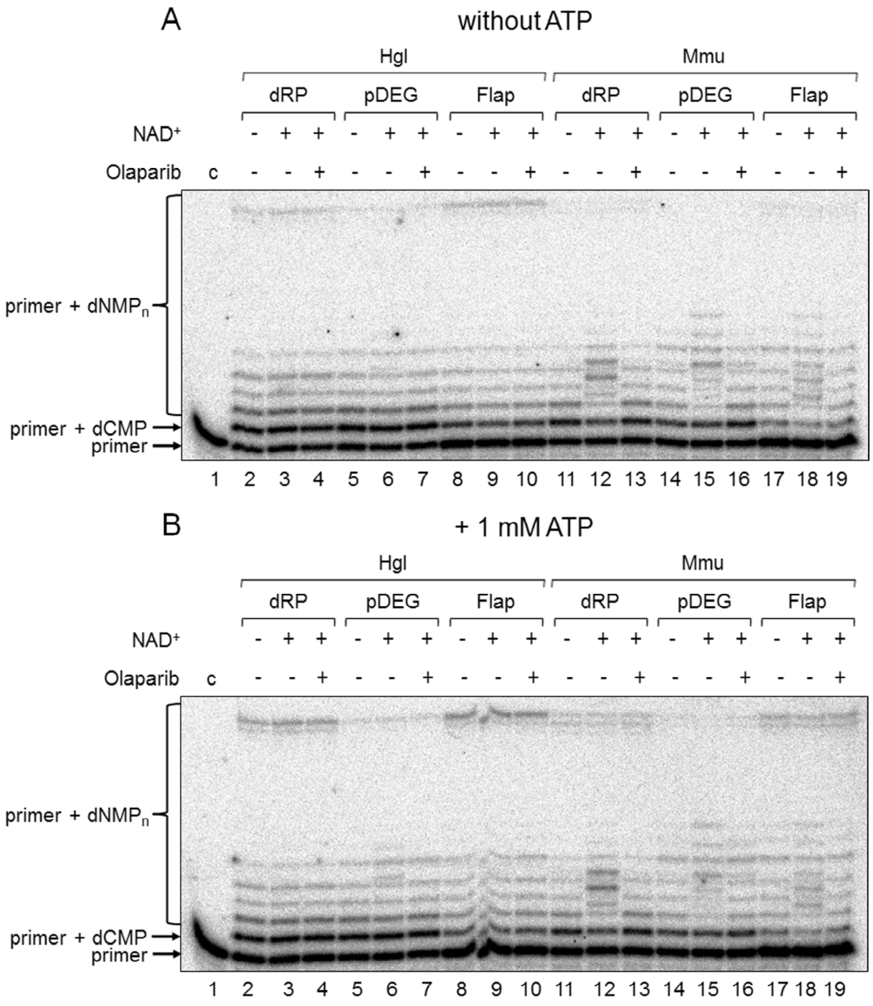

Figure S3. of ATP and olaparib on DNA synthesis in Hgl and Mmu WCEs. Hgl and Mmu cell extract proteins (0.5 mg/ml) were incubated at 37 °C for 5 min with 100 nM DNA duplexes dRP, pDEG, and Flap and 0.1 mM dNTPs in the presence (**B**) or absence (**A**) of 1 mM ATP as described in the section ‘DNA synthesis assay’. 0.5 mM NAD$^+$ and 10 μM olaparib were initially added to the reaction mixtures where indicated. Lane 1 corresponds to the Up oligonucleotide alone (control). The types of DNA substrates and cell lines are indicated.

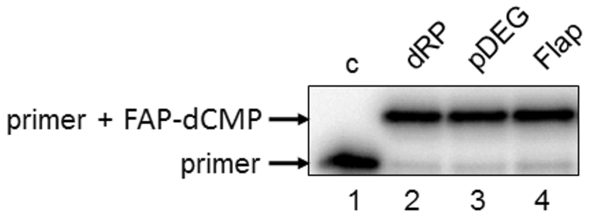

Figure S4. Elongation of primers by Polβ using FAP-dCTP as a substrate during synthesis of photoreactive DNA. Reaction mixtures containing 100 nM 5′-[^{32}P]-labeled DNA substrate, 50 μM FAP-dCTP, and 100 nM Polβ were incubated at 37 °C for 30 min, and then 20 mM EDTA was added to bind Mg^{2+} ions and inactivate Polβ. The types of DNA substrates are indicated.

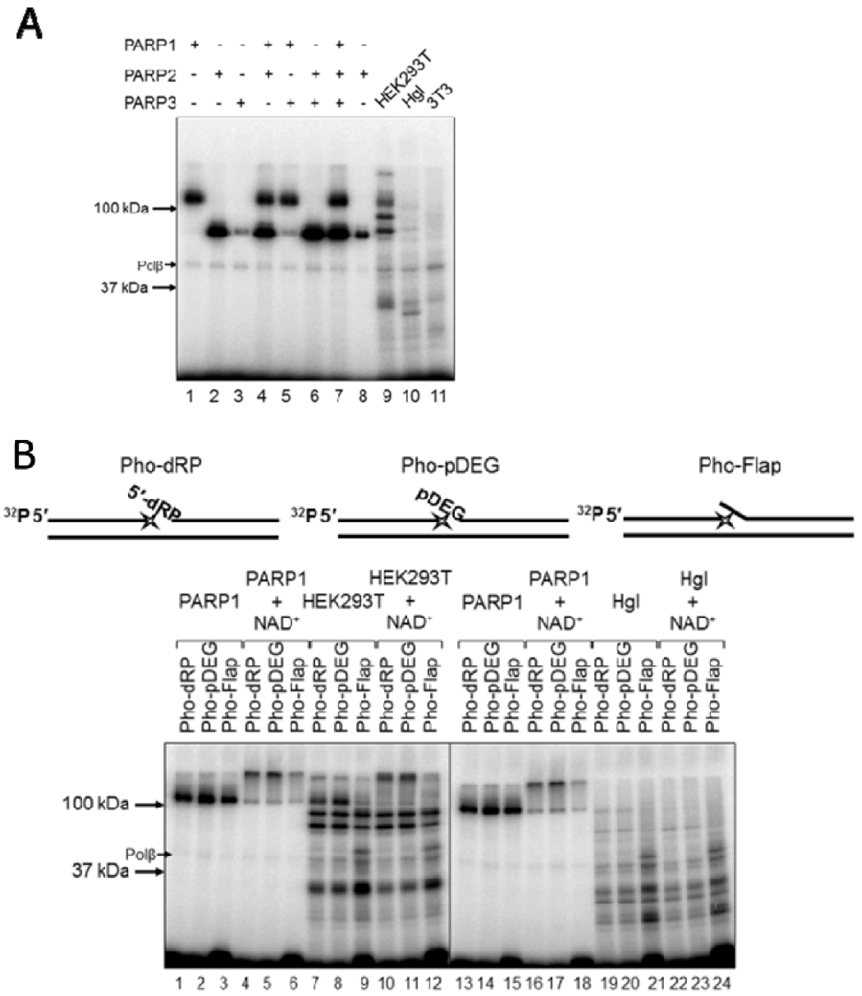

Figure S5. Affinity modification of PARPs and cell extract proteins with photoreactive DNA probes. (**A**) Interaction of PARPs and cell extract proteins with DNA duplex Pho-dRP. Photoaffinity modification was performed as described in the section 'Photoaffinity modification of proteins' using 100 nM DNA duplex Pho-dRP and 1 mg/mL cell extract proteins (HEK293T, lane 9; Hgl, lane 10; 3T3, lane 11), as well as different combinations of purified PARP1 (100 nM, lanes 1, 4, 5, 7), PARP2 (100 nM, lanes 2, 4, 6, 7), and PARP3 (300 nM, lanes 3, 5, 6, 7). Lane 8 corresponds to 1/3 V of the reaction mixture identical to the one loaded on lane 2. The proteins were separated by 12.5% SDS-PAGE and those cross-linked to [^{32}P]-labeled DNA were visualized by autoradiography. (**B**) Influence of PAR synthesis affinity modification of proteins. Affinity modification was performed as described in **A**. Recombinant PARP1 (100 nM, lanes 1–6 and 13–18) or 1 mg/mL cell extract proteins (HEK293T, lanes 7–12; Hgl, lanes 19–24) in the absence (lanes 1–3, 7–9, 13–15, and 19-21) or presence (lanes 4–6, 10–12, 16–18, and 22–24) of NAD^+. The structures of the photoreactive DNAs are schematically shown at the top. The asterisk denotes the FAP-dCMP residue.
